# Research on the impacts of digital economy on carbon emission efficiency at China’s City level

**DOI:** 10.1371/journal.pone.0308001

**Published:** 2024-09-06

**Authors:** Jie Hou, Weidong Li, Xuanhao Zhang

**Affiliations:** 1 School of Business, Liaocheng University, Liaocheng Shangdong, People’s Republic of China; 2 School of Economics and Management, Beijing Jiaotong University, Beijing, People’s Republic of China; 3 School of Economics, Fujian Normal University, Fuzhou, Fujian, People’s Republic of China; Universiti Malaya, MALAYSIA

## Abstract

As a new type of economic format, digital economy has three major characteristics: technical, innovative, energy-saving and environmentally friendly. Acting on various sectors of the national economy, it is beneficial for improving carbon emission efficiency and is of great significance for achieving China’s two major goals of carbon peak and carbon neutrality. Firstly, theoretical analysis of the impact mechanism of digital economy on carbon emission efficiency, proposing research hypotheses on the direct effect, mediating effect, and spatial effect of digital economy on carbon emission efficiency. Secondly, based on panel data from 279 cities in China from 2011 to 2020, the econometric models are constructed to empirically analyze the direct, mediating, and spatial effects of digital economy on carbon emission efficiency. The results show that: 1) Digital economy can improve carbon emission efficiency; 2) The impact of digital economy on carbon emission efficiency has a “U”-shaped relationship, which is consistent with the "Environmental Kuznets Curve" hypothesis; 3) The impacts of digital economy on carbon emission efficiency exist in urban heterogeneity, specifically manifested as regional heterogeneity and urban scale heterogeneity; 4) Technological innovation is an important mediator for improving carbon emission efficiency in digital economy, and promoting technological innovation in digital economy can improve carbon emission efficiency; 5) Digital economy has spatial effect on carbon emission efficiency, which can improve the carbon emission efficiency of neighboring cities. Finally, based on the above results, suggestions are proposed from three aspects: promoting important industries and key areas for deep cultivation of carbon emission in digital economy, emphasizing regional balance in the development of digital economy, and strengthening regional cooperation in the development of digital economy, in order to continue to play a positive role in improving carbon emission efficiency through digital economy.

## Introduction

In recent years, digital economy has developed rapidly in China. According to the Digital China Development Report (2022) released by the China National Internet Information Office, the scale of China’s digital economy will reach 50.2 trillion CNY in 2022, with the total volume firmly ranking second in the world; it will grow by 10.3% year-on-year in nominal terms, and its share of GDP will rise to 41.5%. Digital economy in China has grown into another major economic form after the agricultural and industrial economies, promoting profound changes in the mode of production, lifestyle and governance. At the same time, on the basis of its own actual development, the Chinese Government following the objective laws of economic development, adhering to the concept of high-quality development, and shouldering its responsibility as the world power, has put forward the goal of realizing carbon peak by 2030 and carbon neutrality by 2060. Among them, carbon peak refers to the realization that the total amount of carbon dioxide emissions reaches a historical peak and then stops growing, and gradually decreases after the overall leveling off. Carbon neutrality means that enterprises, organizations or individuals measure the total amount of greenhouse gas emissions directly or indirectly generated, and offset their own carbon dioxide emissions by planting trees, saving energy and reducing emissions, so as to achieve zero carbon dioxide emissions. Carbon peak is the foundation and premise of carbon neutrality, and carbon neutrality can only be achieved by realizing carbon peak; carbon neutrality is a tight constraint on carbon peak, and the time and peak level of carbon peak should be determined under the constraints of the vision of carbon neutrality. The report of the 20th the Communist Party Congress of China in 2022 emphasized that promoting the greening and decarbonization of economic and social development is the key link in realizing high-quality development. Therefore, the development process of digital economy coincides with the process of realizing the goal of carbon neutrality, and it is of practical importance to explore the various impacts of digital economy on carbon emission and its specific paths, so that China can reduce its carbon emission and improve its carbon emission efficiency through digital economy, which is expected to realize the goal of carbon neutrality and to practice high-quality development.

## Literature review

The rapid development of digital economy has had a profound impact on all sectors of the national economy, and the two elements of digital economy—digital technology and data resources, are conducive to reducing carbon emission and improving carbon emission efficiency, thus practicing the strategic goal of carbon peak and carbon neutrality. Existing literature has analyzed the impacts of digital economy on carbon emission from both theoretical and empirical aspects.

On the theoretical aspect, Zhong et al. argued that digital economy, with digitized knowledge and information as its core elements, can reduce energy intensity and enhance ecological efficiency, and the combination with the real economy takes into account the dual demands of transformation and innovation, is a brand-new approach to transforming the region into a low-carbon economy [1]. Lyu et al. argued that it is possible to reduce the proportion of fossil energy by promoting research and development of clean energy with the help of digital economy. At the same time, it can optimize the factor inputs, effectively alleviate the distortion of the energy market, and achieve the decline of the total amount of carbon emission [2]. Wang et al. argued that, on the production aspect, as a result of the development of digital technology, clean or low-carbon production technologies are spreading and being applied in industrial settings, which reduces carbon emission; on the demand aspect, new digital technologies are widely used, reduce the transaction costs of consumers in obtaining information and arriving at deliveries, and reduce or avoids unnecessary offline consumption activities, thus contributing to the reduction of carbon emission [3]. Wang et al. argued that, with the factors integration and the precise matching, digital economy can improve production efficiency, drive industrial optimization, and form a multiple governance system led by the government, enterprises and the public to achieve shared network economies of scale to promote green development and reduce carbon emission in neighboring regions [4]. Hu argued that the development of digital economy realizes the expansion of the service industry and reduces the impact of the industrial production on the environment, and at the same time improves the rapidity of the public service and promotes the popularization of environmental protection technology to reduce the carbon emission of the society [5]. Dong et al. took the reform of e-commerce pilot cities as a detailed study of digital economy, and find that digital economy can form a crowding-out effect on energy-consuming industries, accelerate technological overflow, enhance the accumulation of capital and labor, increase factor utilization efficiency and reduce carbon emission [6].

On the empirical aspect, the existing literature analyzes the impacts of digital economy on carbon emission at the world level and the Chinese level. At the world level, Dong et al. empirically examined the effects of digital economy development on carbon emission and concluded that the development of digital economy significantly reduces carbon intensity, but contributes to an increase in per capita carbon emission, and increases differences in the development of digital economy across countries, which are most pronounced between "hyper-digitalized" and "unconnected" countries [7]. Ma and Zhang examined the impacts of national digitization on fossil fuel efficiency using the System-GMM method, concluding that the impact of digitization on optimal fossil fuel consumption is related to income; rich Asian countries show a positive correlation between digitization and fuel efficiency, carbon emission can be reduced by developing digital economy [8]. At the Chinese level, Zhang et al. used the general regression method to study the direct effect of digital economy on low-carbon development, and then applied the mediating effect model to explore its indirect transmission mechanism, and concluded that digital economy has become an important engine for driving low-carbon development, and can promote low-carbon development through environmental governance, technological innovation, and industrial structure upgrading, in which the mediating role of industrial structure upgrading is the most significant. Meanwhile, the results of regional heterogeneity analysis show that compared with the central and western regions, digital economy has a more significant effect in driving low-carbon development in the eastern region of China [9]. Wang et al. estimated the relationship between digital economy and carbon emission by using the System-GMM method, and affirms the carbon dioxide emission reduction effect of digital economy, while the four major sub-indicators constructed in the article, such as infrastructure, economic growth, can produce carbon emission reduction, which is specifically realized through the reduction of the scale of polluting production, technological innovation and other channels [10]. Wang and Guo used the spatial econometrics model and concluded that the continued expansion of digital financial inclusion can mitigate carbon emissions in cities; digital financial inclusion reduces carbon emissions more in developed and highly industrialized regions than in less developed and lowly industrialized regions [11]. Ma et al. studied the long-term synergistic integration relationship among digitalization, carbon emission, and R&D investment. It concluded that provinces can curb carbon emission through digital transformation, and R&D investment not only suppresses the level of carbon emission, but also serves as a mediator in the correlation between digitalization and carbon dioxide emissions [12]. Chen et al. using a two-way fixed effect model, divided digital economy into three first-level indicators: digital industrialization, industrial digitization, and digital infrastructure to explore the impact of digital economy on carbon emission in detail, and it is found that digital industrialization has the most obvious effect on the carbon emission reduction, and at the same time, the governmental support as a moderating variable is also the most significant between digital industrialization and carbon emission [13]. Wang and Li studied the impacts of digital economy on carbon emission reduction through the two-way fixed effect model and the mediating effect model, and the results concluded that the use of digital economy greatly supported carbon emission reduction, but at the same time limited the growth of both the size and intensity of carbon emission; the effect of digital economy to reduce carbon emission includes the heterogeneity of digital economy dimension, the heterogeneity of industry, the heterogeneity of production and living, and the heterogeneity of urban and rural areas [14]. Bai et al. used the spatial panel smooth transition threshold model to investigate the impacts of digital economy on carbon emission intensity, and found that there is an inverted U-shaped trend in both the direct and spatial impacts of digital economy on carbon intensity, with the threshold node of the trend being influenced by the application of digital technologies and governmental macro-policy regulation; and the effects of digital economy on the intensity of carbon emission are heterogeneous across the various sectors of industry [15]. Zhu and Lan utilized panel data of 277 Chinese cities from 2011–2019 to explore the formation mechanism of carbon rebound effect in Chinese cities through improved the stochastic frontier analysis model, and concluded that digital economy induces and expands Chinese urban carbon rebound effect by increasing energy consumption, thereby inducing and extending the carbon rebound effect in urban areas [16]. Wang and Chen adopting the two-tier stochastic frontier analysis method, the spatial econometric model and the logarithmic mean divisia index method, concluded that the clustering of digital economy in a region and its diffusion to other regions can reduce carbon emission, while reducing energy intensity and the size of population clusters are the main channels through which digital economy can reduce carbon emission [17]. Jun et al. constructed the spatial econometric model to investigate the impacts of digital economy on the efficiency of urban industrial carbon emission, and concluded that the efficiency of industrial carbon emission in Chinese cities has the characteristics of spatial agglomeration; there exists a “U”-shaped nonlinear relationship between digital economy and the efficiency of urban industrial carbon emission [18]. Cheng et al. explored the spatial spillover effect and threshold effect of digital economy on carbon emission intensity, and concluded that while digital economy reduces carbon intensity directly, it also reduces it indirectly through two channels: technological innovation and industrial structure upgrading; there is an inverted “U”-shaped trend in the direct impact of digital economy on carbon intensity, and a “U”-shaped trend in spatial spillovers [19]. Li et al. simplified and processed 51 input-output model sectors of China, and empirically analyzed the impact of China’s digital economy on carbon emission and its evolution through the multi-layer nested method, and concluded that the implied carbon emission of digital economy are greater than its direct carbon emission; the carbon emission from China’s digital economy tend to be concentrated in the energy-intensive upstream and the digital-intensive downstream of the industrial chain [20].

In summary, the existing literature has theoretically discussed the mechanism of digital economy’s impacts on carbon emission, emphasizing that the characteristics of digital economy itself plays vital roles in economic operation, reducing total carbon emission and improving carbon emission efficiency by means of technical support and data utilization. The empirical evidence demonstrates that digital economy has various effects on intensity and efficiency of carbon emission, including direct effect, mediating effect, threshold effect and spatial effect. However, the existing literature has the following shortcomings: 1) The mechanism of the impacts of digital economy on carbon emission efficiency is not sufficient, and the theoretical mechanism and research hypotheses of the direct effect, mediating effect, threshold effect, and spatial effect of digital economy on carbon emission efficiency have not been systematically put forward; 2) China’s territory is vast, there are obvious differences among regions in economic development, it is necessary to examine the regional heterogeneity of digital economy’s impact on carbon emission efficiency, which will help the governments of different regions to implement policies based on their own realities. Meanwhile, China high-quality development pays more attention to improving carbon emission efficiency, to minimize carbon emission while producing the same amount of products or providing the same services. It not only meets the actual demand of China’s long-term economic development, but also is an important approach to realize the goal of carbon neutrality. This paper focuses on the construction of various impacts of digital economy on carbon emission efficiency and its corresponding theoretical mechanisms, and constructs the econometric models for empirical analysis using data from 279 Chinese cities from 2011 to 2020 as samples, to verify the existence of direct effect, nonlinear effect, heterogeneous effect, mediating effect and spatial effect of digital economy on the carbon emission efficiency, guiding the Chinese Government to take measures to seize the opportunity of digital economy, fully utilize digital economy to achieve the targets of carbon peak and carbon neutrality.

## Theoretical mechanism

### Direct effect of digital economy on carbon emission efficiency

Digital economy has two major elements: digital technology and data resources, which can change the way of production and increase the productivity of enterprises from the supply side, and change the consumption mode and accurately connect with customer resources from the demand side, thus reducing the carbon emission per unit of product or service and enhancing the carbon emission efficiency [21]. On the one hand, digital technology has caused a reconstruction in the paradigm of industrial organization. Digital technology has prompted the transformation of industrial organization division to networking, platformization and openness, served the digital, networked and intelligent transformation of enterprises, optimized the production process, and made the form of industrial organization brand new. Digital technology can not only directly improve energy utilization efficiency; even fundamentally changing the traditional energy consumption based production model, the production process relies more on technological investment, and products and services focus more on core technologies. New products and services have replaced low-end products in the market, gradually changing the focus of consumers on products. On the other hand, data resources as input elements have the characteristics of massive, reproducible, shareable and unlimited growth [22]. It can not only partially replace traditional energy. Moreover, applying it to the production and circulation processes can directly reduce production and transaction costs, improve the operating efficiency of the industrial chain and the effect of resource allocation, it also stimulates the technological innovation in the industrial chain, supports the extension of the enterprise focus to the upstream R&D and design links and the downstream service branding links, and reduces the demand for energy; data resources can be used to analyze consumer preferences and directly target audience groups, reducing carbon emission in downstream processes such as distribution and allocation; all of which can enhance the carbon emission efficiency. Here, hypothesis 1 is proposed:

H1: Digital economy can improve carbon emission efficiency directly with the two elements of digital technology and data resources.

At the same time, it has been pointed out in the literature that the impact of digital economy on carbon emission efficiency may have a “U”-shaped nonlinear relationship, which is in line with the hypothesis of the "Environmental Kuznets Curve", that is, the development of digital economy will lead the efficiency of carbon emission to decline first; after crossing the inflection point, the efficiency of carbon emission will rise. In this paper, we believe that it’s because digital economy is still in a rapid development stage, and its impact on various sectors of the national economy needs a certain period of integration before it can play a role. At the early stage of the development of digital economy, in order to adapt to the new paradigm of economic development, the various sectors of the national economy will be free from the original production paradigm and frequently carry out innovation in economic activities, which will lead to an increase in carbon emission and a reduction in carbon emission efficiency; in the middle and late period of digital economy, with the maturity of digital economy, it will exert great impact on national economy, which will enhance the carbon emission efficiency. Here, hypothesis 2 is proposed:

H2: The impact of digital economy on carbon emission efficiency has a “U”-shaped relationship. In the early stage of the development of digital economy, carbon emission efficiency will be reduced; in the middle and late stages of the development of digital economy, carbon emission efficiency will be increased.

Further, there is a general urban heterogeneity in the impact of digital economy on carbon emission efficiency. Especially for China, with a vast land area, subject to the obvious differences in the level of economic development and industrial structure between cities, the impact of digital economy on the efficiency of carbon emission varies significantly from place to place. Considering the level of economic development, digital economy in developed regions starts early, develops rapidly, and has a large scale, which has a large effect on improving carbon emission efficiency; while digital economy in less developed regions starts late, develops relatively slowly, and has a small scale, the improving effect of digital economy is less obvious. Considering the industrial structure, industry is often characterized by heavy pollution and high resource consumption, the more developed the industry is, the larger the carbon emission are, and the more significant the impact of digital economy on carbon emission is. Here, hypothesis 3 is proposed:

H3: There is urban heterogeneity in the impact of digital economy on carbon emission efficiency. The more developed the city’s economy is, the greater the role of digital economy in improving carbon emission efficiency. The more developed the city’s industry is, the greater the role of digital economy in enhancing carbon emission efficiency.

### The mediating effect of digital economy on carbon emission efficiency

Digital economy has two major production factors, digital technology and data resources, which are integrated into various sectors of the national economy, making technological innovation less costly, less time and space constraints, and more active; technological innovation improves the old "input-output" production mode, empowers the efficient operation of various sectors of the industrial chain, and can enhance the carbon emission efficiency. At the same time, relying on digital technology and data resources to establish the platform economy and sharing economy of the industry chain, which makes positive externalities between enterprises, accelerating knowledge exchange and technological innovation between industry chain sectors, strengthening the synergistic relationship between industry chain sectors, and promoting the higher efficiency of the industry chain’s operation, which also enhances the carbon emission efficiency [23]. Here, hypothesis 4 is proposed:

H4: There is a mediating effect of technological innovation in the impact of digital economy on carbon emission efficiency. Digital economy can promote technological innovation, and then technological innovation can enhance carbon emission efficiency.

### Spatial effect of digital economy on carbon emission efficiency

The spatial effect of digital economy on carbon emission efficiency is manifested in the fact that digital economy promotes the spatial separation of various sectors of the industrial chain, and the geographic and administrative boundaries are broken, which makes the technology and knowledge overflow characterized by spatial dissemination, and the communication and interaction between cross-regions become increasingly active, which significantly affects the technological progress of the neighboring regions, and promotes the enhancement of the efficiency of the carbon emission in the neighboring regions [24]. Informative communication methods and openness and sharing of data resources evolved by digital technology break through the time and space limitations, making the technology and knowledge dissemination widespread; the formation of virtual industrial clusters makes the industrial chain division between regions gradually clear, and easily affects the economic activities of neighboring regions; thus, technological innovations flow along the industrial chain, and enhance the carbon emission efficiency of the neighboring regions. At the same time, the continuous development of economy and society also strengthens the connection between cities, and the gradual breaking up of administrative division and the steady improvement of market integration level provide realistic possibilities for the utilization of digital platforms and the sharing of data resources. On this basis, the spatial spillover of knowledge and technology can be successfully realized. Here, hypothesis 5 is proposed:

H5: Digital economy accelerates the cross-regional dissemination of technology and knowledge, and there is a spatial effect on the impact of carbon emission efficiency, which can enhance the carbon emission efficiency of neighboring regions.

**Fig 1:**
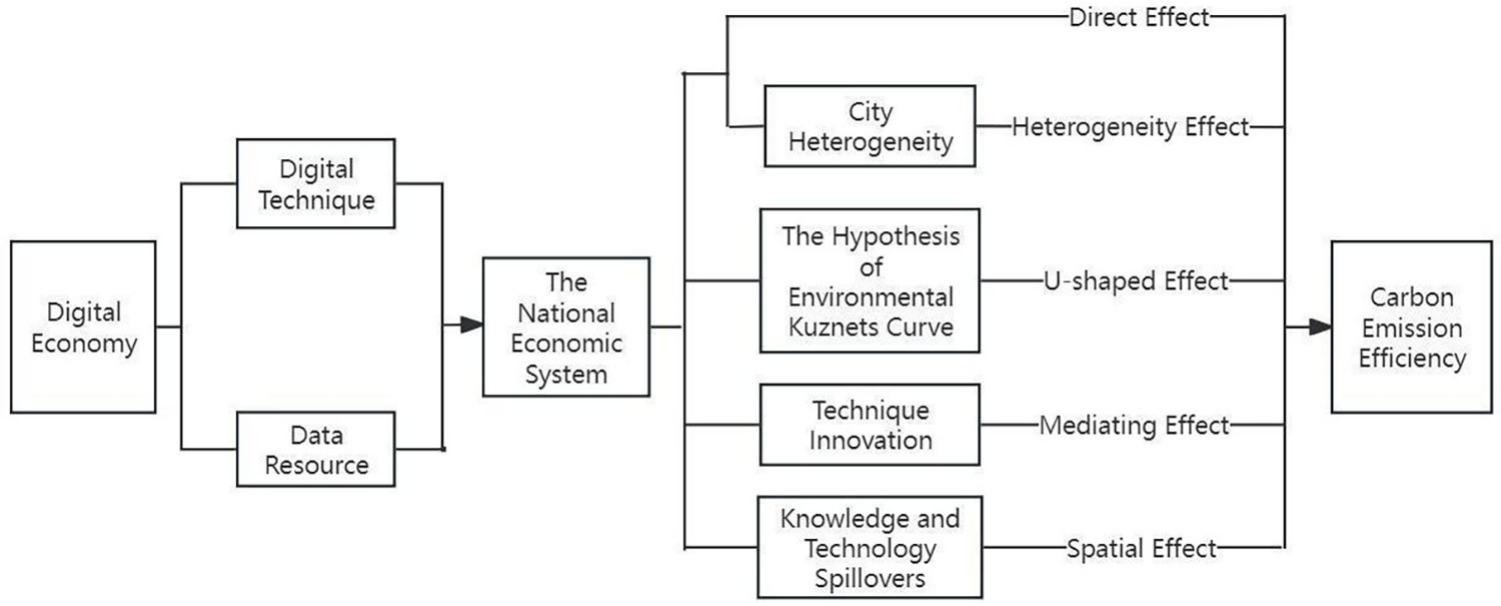
Theoretical framework diagram of the impacts of digital economy on carbon emission efficiency.

In conclusion, the various effects of digital economy on carbon emission efficiency and its theoretical mechanisms proposed in this paper are shown in Fig 1.

Fig 1 shows the specific paths through which digital economy has various impacts on carbon emission efficiency. Digital economy possesses two major production factors, namely digital technology and data resources, which have the significant advantages of technology, innovation and energy conservation and environmental protection, and have profound changes in the economic operation mode from the supply side and the consumption side, which in turn improve carbon emission efficiency. At the same time, the impact of digital economy on carbon emission efficiency is not single, there are direct effect, “U”-shaped nonlinear relationship, heterogeneity effect, mediating effect and spatial effect and other effects. Therefore, the systematic analysis of the impact of digital economy on carbon emission efficiency is significant for understanding the concrete role of digital economy in reducing carbon emission, and it is a solid basis to enrich digital economic theory, carry out empirical analyses and propose policy recommendations.

## Empirical analysis

Based on the above theoretical mechanism of the impacts of digital economy on carbon emission efficiency and its research hypothesis, this paper constructs the corresponding econometric models for empirical analysis, focusing on the direct, mediating and spatial effects of digital economy on carbon emission efficiency in China.

### Modeling

1) The panel regression model constructed in this paper is as follows:

$${CEE}_{it}=c+\alpha {DIGE}_{it}+\beta_{1}{lnPGDP}_{it}+\beta_{2}{STRU}_{it}+\beta_{3}{GOV}_{it}+\beta_{4}{HUM}_{it}+\mu_{i}+\delta_{t}+\varepsilon_{it}$$ (1)

The statistical assumptions of the regression models are four points, namely Linear assumption; Strict exogeneity; There is no strict multicollinearity; Spherical perturbation term. ①Linear assumption. The marginal effect of the explanatory variable on the dependent variable is constant. ②Strict exogeneity. The expected condition for the perturbation term is 0. ③There is no strict multicollinearity. Full rank data matrix. ④Spherical perturbation term. The perturbation term satisfies the properties of homoscedasticity and no autocorrelation.

Further, in order to examine whether there is a nonlinear relationship between the impact of digital economy on carbon emission efficiency, add (DIGE)^2^ constructed the panel regression model as shown below:

$${CEE}_{it}=c+\alpha {DIGE}_{it}+\varphi {\left(DIGE\right)}^{2}+\beta_{1}{lnPGDP}_{it}+\beta_{2}{STRU}_{it}+\beta_{3}{GOV}_{it}+\beta_{4}{HUM}_{it}+\mu_{i}+\delta_{t}+\varepsilon_{it}$$ (2)

where CEE denotes carbon emission efficiency, DIGE denotes the level of digital economy development, PGDP denotes the level of economic growth, STRU denotes the level of industrial structure, GOV denotes the level of government macro-control, HUM denotes the level of human capital, and c denotes a constant term. i denotes the city and t denotes the year. μ_i_ and δ_t_ represent the fixed effects of controlling cities and years. ε_it_ denotes random error term, ε_it_~N(0,*σ*^2^).

2)The mediating effect recursive equation constructed is shown below:

$${CEE}_{it}=c_{1}+\alpha {DIGE}_{it}+\delta Z_{it}$$

$${TECH}_{it}=c_{2}+\beta {DIGE}_{it}+\theta Z_{it}$$

$${CEE}_{it}=c_{3}+\gamma {TECH}_{it}+\mu {DIGE}_{it}+\vartheta Z_{it}$$ (3)

Where CEE denotes carbon emission efficiency, DIGE denotes the level of digital economy development, and TECH denotes the level of technological innovation; Z_it_ denotes the control variable, and c denotes the constant term. i denotes the city and t denotes the year.

3) The spatial econometrics model includes the Spatial Lag Model (SLM), the Spatial Error Model (SEM) and the Spatial Durbin Model (SDM), and the spatial econometrics model constructed in this paper is shown below:

$${CEE}_{it}=\rho W{CEE}_{it}+\beta X_{it}+\theta WX_{it}+\varepsilon_{it}$$

$$\varepsilon_{it}=\lambda W\varepsilon_{it}+\mu_{it}$$ (4)

Where CEE denotes carbon emission efficiency, X denotes the independent variable DIGE and the control variable Z. β denotes the regression coefficient of X, and ρ、θ、λ denotes the spatial correlation coefficient. ε_it_denotes the random perturbation term. i denotes city and t denotes year. μ_i_ and δ_t_ represent the fixed effects of controlling cities and years. ε_it_ denotes random error term, ε_it_~N(0,σ^2^).Then there are:

Ifρ≠0,θ=λ=0,thenconsistentwiththeSLM

Ifρ=0,θ=0,λ≠0,thenconsistentwiththeSEM

Ifρ≠0,θ≠0,λ=0,thenconsistentwiththeSDM (5)

W denotes the spatial weight matrix. In this paper, the spatial weights matrices based on geographic distance 1/d_ij_ is used for the measurement, which denotes the computation of the spherical distance between city i to city j based on the city’s position in latitude and longitude. Its advantage is that it will not change with the change of transportation speed and the change of sample time length, and has strong robustness.

Meanwhile, the spatial econometrics model needs to be based on spatial correlation. Spatial auto-correlation is divided into global spatial auto-correlation and local spatial auto-correlation, and this paper adopts the global moran’s I (MI) and local moran’s I (LMI) to measure respectively, and its calculation formula is shown below:

$$MI=\frac{n\sum_{i=1}^{n}\sum_{j=1}^{n}W_{ij}(x_{i}-\bar{x})(x_{j}-\bar{x})}{\sum_{i=1}^{n}{\left(x_{i}-\bar{x}\right)}^{2}\sum_{i=1}^{n}\sum_{j=1}^{n}W_{ij}}$$ (6)

$$LMI=\frac{n(x_{i}-\bar{x})}{\sum_{i=1}^{n}{\left(x_{i}-\bar{x}\right)}^{2}}\sum_{j=1}^{n}W_{ij}(x_{j}-\bar{x})$$ (7)

Where x denotes the carbon emission efficiency and W_ij_ denotes the spatial weights matrices based on geographic distance.

### Variable selection

Dependent variable: Carbon Emission Efficiency (CEE). Drawing on the method of Tone [25], it is calculated by using real fixed capital stock, total number of employees, and total electricity consumption as inputs, real GDP as desired outputs, and carbon dioxide emissions as non-desired outputs using the Super-SBM Model. Among them, fixed capital stock and GDP are deflated based on the year of 2011 by fixed asset price index and GDP index to obtain the real values, get the real fixed capital stock and real GDP.

Independent variable: The Level of Digital Economy Development (DIGE). Drawing on the ideas of Jie Wu et al., the evaluation index system of level of digital economy development is constructed as shown in Table 1 [6], and the entropy weight method is used to calculate the level of digital economy development.

**Table 1:** Evaluation index system for the level of digital economy development.

Target level	Level 1 indicators	Level 2 indicators	Indicator properties
Level of digital economy development	Internet penetration	Internet users per 100 population	forward
	Number of Internet-related employees	Percentage of employees in the computer and software industry	forward
	Internet-related outputs	Telecommunications services per capita	forward
	Number of mobile Internet users	Cell phone subscribers per 100 population	forward
	Level of development of digital inclusion	Peking University Digital Inclusive Finance Index	forward

Mediating variables: The Level of Technological Innovation (TECH), measured using the number of patents granted (ten thousand). The number of patents granted is the effective indicator for evaluating technological innovation [26].

Control variables: 1) the Level of Economic Growth (lnPGDP), measured using the logarithm of per capita GDP (10,000 CNY). Economic growth can provide material foundation and technological support for improving carbon emission efficiency [27]. 2) the Level of Industrial Structure (STRU), measured using the ratio of tertiary industry output to secondary industry output. The transformation of industrial structure can directly affect the changes in carbon emission efficiency [28]. 3) the Level of Government Macro-Control (GOV), measured using the ratio of the amount of local general public financial budget expenditure to GDP. The government formulates policies to regulate the reduction of total carbon emissions and the improvement of carbon emission efficiency in the whole society [29]. 4) the Level of Human Capital (HUM), measured by the ratio of the number of students enrolled in general higher education institutions to the resident population. Human capital is a necessary condition for technological progress to promote carbon emission efficiency improvement [30].

### Data sources

This paper selects the panel data of 279 Chinese cities from 2011 to 2020, and most of the relevant data of each variable come from China Urban Statistical Yearbook, while a few missing values come from the statistical yearbooks of each city and the annual work report of the government or are made up by interpolation. The data descriptive statistics for each variable are shown in Table 2.

**Table 2:** Data descriptive statistics.

	Sample size	Mean	Standard deviation	Minimum	Maximum	1/VIF
*CEE*	2790	0.4211	0.1436	0.1481	1.1931	—
*DIGE*	2790	0.2023	0.0961	0.0283	0.8578	0.1477
*TECH*	2790	0.6112	1.4706	0.0009	22.2412	0.3464
Ln*PGDP*	2790	1.5026	0.5641	-0.4374	3.2460	0.2628
*STRU*	2790	1.0222	0.5577	0.1750	5.3482	0.5278
*GOV*	2790	0.1949	0.0858	0.0439	0.6576	0.4657
*HUM*	2790	0.0177	0.0205	0.0001	0.1256	0.6396

From Table 2, it can be seen that the basic information of the meanings, maximum values, mean values, and standard deviations of each variable mainly reflects the centralized and discrete trends of the data, which are consistent with the reality and no outliers were found. Meanwhile, the VIF test results show that the correlation between variables is not strong.

### Analysis of empirical results

In this paper, the constructed econometric models are regressed and analyzed using STATA15.0 software, and the results are as follows.

#### The results and analysis of the panel regression model

Using the ordinary least squares regression method for estimation, the regression results of the direct effect and nonlinear relationship of the impacts of digital economy on carbon emission efficiency are shown in Table 3.

**Table 3:** Results of the ordinary panel regression model.

	(1)	(2)	(3)	(4)
*DIGE*	1.0031*** (11.01)	0.0994 (0.53)	0.9830*** (11.25)	-0.0808 (-0.45)
(*DIGE*)^2^	—	0.8335*** (5.47)	—	0.9835*** (6.76)
*Controls*	No	No	Yes	Yes
*Con_*	0.2759*** (24.71)	0.3636*** (18.64)	0.2034*** (7.93)	0.2939*** (10.23)
*City FE*	Yes	Yes	Yes	Yes
*Year FE*	Yes	Yes	Yes	Yes
*R-Square*	0.1297	0.1400	0.2155	0.2296
*F-value*	37.28***	37.00***	48.99***	49.58***

Note: *, **, and *** indicate significant at the 10%, 5%, and 1% levels, respectively, and the values inside the parentheses are t-values, as below.

Table 3 shows that digital economy has a significant positive effect on carbon emission efficiency, indicating that digital economy can improve carbon emission efficiency. At the same time, the influence of digital economy on carbon emission efficiency has a “U”-shaped relationship. At the early stage of digital economy development, digital economy as a new economic mode into the national economy sectors, corresponding to the increase of carbon emission in various sectors, also need a certain period of transition to change the production paradigm and optimize the organization form, and will reduce the carbon emission efficiency; as digital economy matures into the middle and late stages of development, it has a significant positive impact on all segments of the national economy, which will enhance the efficiency of carbon emission [31].

Further, this paper divides 279 cities into three groups of eastern, central and western cities according to China’s provincial division method, and into four groups of mega-cities, large cities, medium cities, and small cities according to the resident population to examine the urban heterogeneity of digital economy on the carbon emission efficiency [Note]. Using the ordinary least squares regression method for estimation, the regression results are shown in Table 4.

**Table 4:** Results of urban heterogeneity.

	Eastern cities	Central cities	Western cities	Mega-cities	Large cities	Medium cities	Small cities
*DIGE*	0.6302*** (4.84)	0.9957*** (6.19)	0.6821*** (3.10)	0.7988*** (3.13)	0.4414*** (2.91)	0.4340* (1.65)	-0.2161 (-0.59)
*Controls*	Yes	Yes	Yes	Yes	Yes	Yes	Yes
*Con_*	0.2669*** (5.58)	0.0887** (2.39)	0.2013*** (4.27)	0.1189*** (0.77)	0.2492*** (7.57)	0.3028*** (6.31)	0.2875*** (3.11)
*City FE*	Yes	Yes	Yes	Yes	Yes	Yes	Yes
*Year FE*	Yes	Yes	Yes	Yes	Yes	Yes	Yes
*R-Square*	0.3206	0.3054	0.2026	0.4278	0.2349	0.2295	0.0607
*F-value*	29.56***	30.36***	11.34***	12.71***	28.70***	12.34***	2.73**

As can be seen in Table 4, the heterogeneity of the corresponding cities in the east, central and west of the country is examined from the geographical perspective, and it is found that digital economy of the cities in the east, central and west of the country has a significant positive impact on carbon emission efficiency, which can enhance carbon emission efficiency. Digital economy of cities in the central region has the greatest effect on carbon emission efficiency, followed by cities in the western region, and the smallest is cities in the eastern region. This is due to the fact that with the transformation of the industrial structure of the cities in the eastern region vigorously develop the service industry, the industry moves gradually towards central and western cities, where the industry represents a major source of energy consumption and carbon emission compared to service industry, which makes digital economy more significant in improving the carbon emission efficiency of the industry than that of the service industry. From the perspective of scale, we examine the heterogeneity of mega-cities, large cities, medium cities and small cities, and find that there is an obvious city scale gradient in the size of the role of digital economy in improving carbon emission efficiency. Mega-cities have the largest role, followed by large cities, medium cities, and finally small cities. The larger the city scale, the larger the scale of digital economic development, the more robust, the greater the role of digital technology and data resources in enhancing carbon emission efficiency; the smaller the city scale, the smaller the scale of digital economic development, the weaker the continuity, the smaller the role of digital technology and data resources in enhancing carbon emission efficiency. In conclusion, there is indeed urban heterogeneity in the impact of digital economy on carbon emission efficiency. Due to the fact that industry is the main source of carbon emissions, technological innovation has emerged as the first way in big cities to affect carbon emissions, the more developed the city industry and the larger the city size, the greater the role of digital economy in improving carbon emission efficiency. This also reminds the government to adapt to local conditions and improve policy efficiency in the process of carbon reduction.

#### Regression results and analysis of the mediating effect model

Using the ordinary least squares regression method for estimation, the regression results of technological innovation as the mediating effect of digital economy on carbon emission efficiency are shown in Table 5.

**Table 5:** Results of mediated effects.

	Dependent variable: *CEE*	Mediating variable: *TECH*	Dependent variable: *CEE*
*DIGE*	0.9830*** (11.25)	22.3442*** (33.65)	0.6293*** (6.02)
*TECH*	—	—	0.0158*** (6.05)
*Controls*	Yes	Yes	Yes
*Con_*	0.2034*** (7.93)	-1.8144*** (-9.30)	0.2321*** (8.96)
*City FE*	Yes	Yes	Yes
*Year FE*	Yes	Yes	Yes
*R-Square*	0.2155	0.4299	0.2268
*F-value*	48.99***	134.50***	48.82***

As can be seen from Table 5, when no mediating variable is introduced in the model, digital economy can improve carbon emission efficiency. For every 1% increase in the level of digital economy development, carbon emission efficiency improves by 0.9830%. When examining the effect of the independent variables on the mediating variable, digital economy has a significant positive effect on technological innovation, and for every 1% increase in the level of digital economy development, the level of technological innovation increases by 22.3442%. When the mediating variable is introduced in the model, both digital economy and technological innovation can improve carbon emission efficiency. For every 1% increase in the level of digital economy development, carbon emission efficiency is raised by 0.6293%; for every 1% rise in the level of technological innovation, carbon emission efficiency is raised by 0.0158%. After calculation, the mediating effect of technological innovation accounts for 35.91% of the total effect.

#### Regression results and analysis of the spatial econometric model

**Fig 2:**
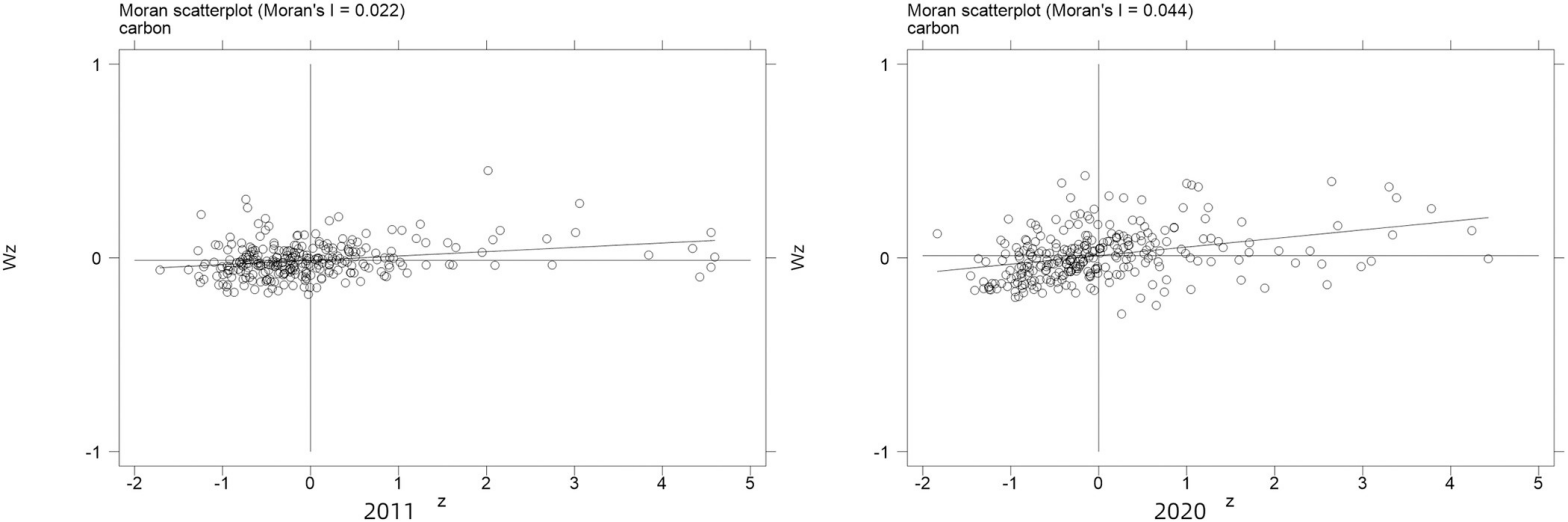
The Scatterplots of local moran’s I for selected years.

First of all, the spatial auto-correlation test is required before conducting the regression of spatial econometric model. The results of global moran’s I and local moran’s I, as shown in Table 6 and Fig 2.

**Table 6:** The results of global moran’s index.

Year	I-value	Z-value	P-value	Year	I-value	Z-value	P-value
2011	0.022	4.202	0.000	2016	0.027	4.997	0.000
2012	0.027	4.872	0.000	2017	0.031	5.522	0.000
2013	0.028	5.031	0.000	2018	0.028	5.124	0.000
2014	0.029	5.195	0.000	2019	0.041	7.182	0.000
2015	0.030	5.361	0.000	2020	0.044	7.716	0.000

Table 6 shows the global moran’s I values of carbon emission efficiency of 279 cities in China calculated by spatial weight matrix based on geographic distance. The global moran’s I values of carbon emission efficiency of 279 cities during 2011–2020 are all positive and pass the significance test at 1% level, indicating that there is some spatial agglomeration of carbon emission efficiency of 279 cities. And the global moran’s I value shows an increasing trend, indicating that the spatial agglomeration of carbon emission efficiency of the 279 cities increases year by year.

The scatterplots of the local moran’s I of carbon emission efficiency of 279 Chinese cities in 2011 and 2020 obtained from the spatial weight matrix based on geographic distance can be seen in Fig 2. The local moran’s I values of most cities are in the HH and LL zones, indicating that the spatial distribution of urban carbon emission efficiency is less different and there is a significant positive spatial correlation.

Secondly, this paper carries out spatial econometric model regression and obtains the results of the SLM, SEM and SDM, as shown in Table 7.

**Table 7:** Results of the spatial econometric model.

	(1)SLM	(2)SLM	(3)SEM	(4)SEM	(5)SDM	(6)SDM
*DIGE*	0.9767*** (11.57)	0.9654*** (11.80)	1.0138*** (11.72)	1.0376*** (12.48)	1.0234*** (11.62)	0.9324*** (11.15)
W*DIGE	—	—	—	—	-1.2704** (-1.82)	-0.3767*** (-4.71)
*Controls*	No	Yes	No	Yes	No	Yes
*City FE*	Yes	Yes	Yes	Yes	Yes	Yes
*Year FE*	Yes	Yes	Yes	Yes	Yes	Yes
*ρ* (λ)	0.8193*** (15.66)	0.6667*** (8.02)	0.8273*** (16.39)	0.8002*** (13.92)	0.8294*** (16.57)	0.6769*** (8.03)
*Sigma* ^2^	0.0049*** (37.21)	0.0046*** (37.24)	0.0049*** (37.21)	0.0045*** (37.22)	0.0049*** (37.24)	0.0044*** (37.24)
*R-Square*	0.1439	0.1808	0.1624	0.2060	0.1705	0.2348
*Log-likelihood*	3436.0246	3555.0708	3437.6219	3572.6535	3437.6697	3588.9404

As can be seen from Table 7, the regression results of the three forms of spatial econometric models can conclude that there is a significant spatial effect of digital economy on carbon emission efficiency. That is, digital economy can not only affect the carbon emission efficiency of the city, but also affect the carbon emission efficiency of neighboring cities.

Finally, the regression coefficients of the spatial econometric model cannot be directly used to measure the impact of digital economy on carbon emission efficiency. Thus, this paper decomposes the effects of the regression results of the spatial econometric models, and since the SEM has no direct and indirect effects, this paper obtains the direct, indirect and total effects of digital economy on carbon emission efficiency of the SLM and the SDM, and the results are shown in Table 8.

**Table 8:** Decomposition of the effects of digital economy on carbon emission efficiency.

*DIGE*	Direct effect	Indirect effect	Total effect
SLM	0.9770*** (11.52)	2.2306* (1.95)	3.2076*** (2.76)
SDM	0.9442*** (10.89)	2.2787** (1.70)	3.2229** (2.36)

As can be seen from Table 8, the direct, indirect and total effects of digital economy on carbon emission efficiency of the SLM and the SDM are all significantly positive, indicating that digital economy of a city not only improves its carbon emission efficiency, but also improves carbon emission efficiency of the neighboring cities, which has a positive externality.

#### Robustness test

In order to verify the robustness of the regression coefficients obtained from the various econometric models above, this paper conducts robustness tests. Among them, the regression results of the panel regression model and the mediating effect model are tested by the variable substitution method, and the regression results of the spatial econometric model are tested by the method of transforming the spatial weight matrix.

In this paper, the calculation method of level of digital economic development is adjusted, still based on the evaluation index system in Table 1, and the Principal Component Analysis(PCA) Method is used to replace the Entropy Weight Method to calculate the level of digital economic development and then carry out the regression of panel regression model, and the results are shown in Table 9.

**Table 9:** Robustness tests for the panel regression models.

	(1)	(2)	(3)	(4)
*DIGE*	0.0708*** (10.30)	0.0336*** (3.22)	0.0721*** (10.96)	0.0306*** (3.08)
(*DIGE*)^2^	—	0.0048*** (4.75)	—	0.0054*** (5.55)
*Controls*	No	No	Yes	Yes
*Con_*	0.4435*** (62.99)	0.4086*** (40.19)	0.3726*** (16.38)	0.3254*** (13.47)
*City FE*	Yes	Yes	Yes	Yes
*Year FE*	Yes	Yes	Yes	Yes
*R-Square*	0.1246	0.1325	0.2136	0.2232
*F-value*	35.61***	34.70***	48.44***	47.80***

Table 9 shows that digital economy has a significant positive effect on carbon emission efficiency, indicating that digital economy can improve carbon emission efficiency. At the same time, there is a “U”-shaped relationship between the effect of digital economy on carbon emission efficiency. This supports the robustness of the regression results in Table 3.

The regression results of the mediating effect model are shown in Table 10.

**Table 10:** Robustness test of the mediating effect model.

	Dependent variable: *CEE*	Mediating variable: *TECH*	Dependent variable: *CEE*
*DIGE*	0.0721*** (10.96)	1.6914*** (33.98)	0.0446*** (5.65)
*TECH*	—	—	0.0163*** (6.20)
*Controls*	Yes	Yes	Yes
*Con_*	0.3726*** (16.38)	2.0531*** (11.92)	0.3391*** (14.61)
*City FE*	Yes	Yes	Yes
*Year FE*	Yes	Yes	Yes
*R-Square*	0.2136	0.4334	0.2255
*F-value*	48.44***	136.43***	48.45***

As shown in Table 10, technological innovation is an important mediator of digital economy to improve carbon emission efficiency. It is calculated that the mediating effect of technological innovation accounts for 61.82% of the total effect. This supports the robustness of the regression results in Table 5.

In this paper, the spatial weight matrix based on geographic distance is converted into adjacency spatial weight matrix for spatial econometric model regression, the results of the direct, indirect and total effects of digital economy on carbon emission efficiency of the SLM and the SDM are shown in Table 11.

**Table 11:** Robustness tests of spatial econometric models.

*DIGE*	Direct effects	Indirect effects	Total effects
SLM	0.9692*** (11.47)	0.1811* (5.16)	1.1502*** (10.88)
SDM	0.9423*** (11.04)	0.2074** (5.69)	1.1497** (10.78)

As can be seen from Table 11, the direct, indirect and total effects of digital economy on carbon emission efficiency are also significantly positive for both the SLM and the SDM. This supports the robustness of the regression results in Table 8.

## Conclusion and recommendation

This paper focuses on examining the various impacts of digital economy on carbon emission efficiency from the city level, in order to lead digital economy to develop well and increase carbon emission efficiency. Theoretically, it builds up various impacts of digital economy on carbon emission efficiency, and points out that digital economy can directly improve carbon emission efficiency; due to the impact of digital economy on carbon emission efficiency, there is a certain period of transition, which makes the impact of digital economy on carbon emission efficiency have a “U”-shaped relationship; Based on the different scale of development and industrial structure of digital economy, there is urban heterogeneity in the impact of digital economy on carbon emission efficiency. The integration of digital technology and data resources into various sectors of the national economy reduces spatial and temporal constraints, and enhances activity level of technological innovation, technological innovation can improve carbon emission efficiency. This indicates that technological innovation has become an important mediator for digital economy to enhance the carbon emission efficiency. Digital economy breaks the spatial and temporal limitations, spreads knowledge and technology across regions, promotes technological progress, and can enhance the carbon emission efficiency of neighboring cities; it shows that there is a spatial effect of digital economy on the efficiency of carbon emission, and it has a positive externality. Empirically, the corresponding econometric models are constructed based on the panel data of 279 Chinese cities from 2011 to 2020 to empirically analyze the various effects of digital economy on carbon emission efficiency, and the results conclude that: 1) Digital economy has a significant positive effect on carbon emission efficiency, and digital economy can enhance carbon emission efficiency. 2) The effect of digital economy on carbon emission efficiency has a “U”-shaped relationship, in line with the "Environmental Kuznets Curve" hypothesis. That is, in the early stage of the development of digital economy, it will reduce the carbon emission efficiency; in the middle and late stage of the development of digital economy, it will enhance the carbon emission efficiency.3) The impact of digital economy on the carbon emission efficiency exists in urban heterogeneity. The effect of digital economy on carbon emission efficiency in central and western cities is greater than that in eastern cities; the effect of digital economy on carbon emission efficiency is characterized by scale hierarchy; the higher the scale hierarchy of the city, the greater the effect of digital economy on carbon emission efficiency. 4) Technological innovation is an important mediator for digital economy to enhance the carbon emission efficiency, and technological innovation promoted by digital economy can enhance the carbon emission efficiency. 5) There are the spatial effects of digital economy on carbon emission efficiency. The development of digital economy in a city can not only improve its own carbon emission efficiency, but also improve the carbon emission efficiency of neighboring cities. Based on the above conclusions, this paper puts forward the following suggestions:

First, China continues to promote the development of digital economy and plow into important carbon-emitting industries and key regions. Through fully utilizing digital technology and data resources, we will, on the one hand, promote the extension of digital economy from the production sector to the living sector and the management sector, so as to balance energy conservation and efficient use of carbon while promoting economic development; on the other hand, invest digital economy in the key carbon-emitting industries and key regions, especially the energy sector, heavy industry and the central region, so as to scale up the effects of digital economy in improving carbon efficiency. In order to achieve the dual goal of carbon peaking and carbon neutrality, digital economy should be fully utilized to further transform the mode of economic development, realize high-quality economic development, and inject new momentum into the green development of the economy and society [32].

Secondly, China should actively promote the transformation of digital achievements, and utilize technological innovation in the practical activities to reduce carbon emission. Technological innovation, as an important mediator of digital economy to improve carbon emission efficiency, should pay attention to the acquisition and transformation of innovation results, in order to continuously improve the carbon emission efficiency, and fundamentally improve the current unfavorable situation of China’s high total carbon emission, and low carbon emission efficiency. Universities, research institutes and enterprises, as the main bodies of science and technology research and development, should continue to tap into the new growth potential of digital economy, develop new products, services and technologies and put them into practice to reduce carbon emission, thereby helping to improve carbon efficiency.

Thirdly, China should promote digital economy nationwide and reduce regional imbalances. Digital economy as a new type of economy, by the capital, technology, talent, infrastructure preferences will be prioritized in the developed cities and under the cumulative effect of the cycle of rapid growth and expansion, while digital economy develops slowly in less developed cities, which results in the disparity between regions is gradually widening. Especially for countries like China, it is a common phenomenon and a practical problem. This requires that, on the basis of respecting the spontaneous mechanism of the market, the Chinese government should actively exert macroeconomic control by adopting fiscal and monetary policy to support less developed cities’ digital economy development, especially in small and medium cities and remote areas in the west, at the same time, it should advance the construction of digital infrastructure and the digital development of industrial chains, and strengthen the cooperation between the eastern and western regions of the digital economy to accelerate the promotion of strengths and weaknesses in the eastern, central, and western regions, promote advanced technology in the eastern region to support the transformation and upgrading of the central and western regions, and create convenience for products from the central and western regions to enter the eastern market, ultimately realizing the sharing of the dividends of digital economic development [33].

Fourth, China should strengthen regional cooperation in the development of digital economy, and exert positive external factors to promote technological innovation to further enhance the carbon emission efficiency. Digital economy has significant knowledge and technology externalities, which can promote the technological progress of neighboring cities and enhance the carbon emission efficiency. Therefore, cities can strengthen digital economy cooperation and establish mechanism for research and development, cooperation and sharing, especially between large cities and small cities, and between eastern, central and western cities, so as to gain the mutual benefits, and to continue to enhance the carbon emission efficiency.

Of course, research on the impacts of digital economy on carbon emission efficiency still requires continuous optimization of methods and enrichment of content by subsequent scholars. Especially with the improvement of the evaluation index system for the digital economy, the optimization of carbon emission efficiency calculation methods, and the application of more advanced econometric models and empirical methods, the effectiveness can be evaluated more comprehensively, guiding government regulation and corporate behavior rationally.

### Notes

Criteria for the division of mainland China regarding eastern, central, and western provinces: the eastern provinces include the 11 provinces of Beijing, Tianjin, Hebei, Liaoning, Shandong, Jiangsu, Shanghai, Zhejiang, Fujian, Guangdong, and Hainan; the central region includes the 9 provinces of Heilongjiang, Jilin, Inner Mongolia, Shanxi, Henan, Anhui, Hubei, Hunan, and Jiangxi; and the western region includes the 11 provinces of Chongqing, Sichuan, Shaanxi, Yunnan, Guizhou, Guangxi, Gansu, Qinghai, Ningxia, Xinjiang, and Tibet. The criteria for classifying the size of cities are as follows: with the permanent population of the urban area as the statistical caliber, a population of less than 500,000 is a small city, a population of between 500,000 and 1,000,000 is a medium city, a population of between 1,000,000 and 5,000,000 is a large city, a population of more than 5,000,000 is a mega-city.

## Supporting information

S1 Data set (XLSX)
